# Fundus Refraction Offset as a Personalized Biomarker for 12-Year Risk of Retinal Detachment

**DOI:** 10.1167/iovs.66.9.1

**Published:** 2025-07-01

**Authors:** Fabian Yii, Ian J. C. MacCormick, Niall Strang, Miguel O. Bernabeu, Tom MacGillivray

**Affiliations:** 1 Robert O. Curle Ophthalmology Suite, Institute for Regeneration and Repair, The University of Edinburgh, Edinburgh, Scotland, United Kingdom; 2 Centre for Clinical Brain Sciences, The University of Edinburgh, Edinburgh, Scotland, United Kingdom; 3 Institute for Adaptive and Neural Computation, School of Informatics, The University of Edinburgh, Edinburgh, Scotland, United Kingdom; 4 Department of Vision Sciences, Glasgow Caledonian University, Glasgow, Scotland, United Kingdom; 5 Centre for Medical Informatics, Usher Institute, The University of Edinburgh, Edinburgh, Scotland, United Kingdom

**Keywords:** fundus refraction offset, myopia, retinal detachment (RD), refractive error, fundus imaging

## Abstract

**Purpose:**

The purpose of this study was to investigate the potential of a novel anatomical metric of ametropia—fundus refraction offset (FRO)—in stratifying the risk of retinal detachment (RD) or breaks, beyond the influence of risk factors including spherical equivalent refraction (SER).

**Methods:**

Participants from the UK Biobank with no prior history of RD/breaks were analyzed (*n* = 9320). The onset of RD/breaks over a 12-year follow-up period was determined based on linked healthcare data. A previously trained deep learning model was applied to each fundus photograph to predict SER. FRO was defined as the error in the fundus-predicted SER, with a negative value indicating a relatively myopic-looking fundus. Cox regression was used to examine the association of baseline FRO with RD/breaks—adjusting for baseline SER, baseline age, sex, and cataract surgery during follow-up. In a subgroup of participants (*n* = 7127) with high-quality optical coherence tomography scans, we additionally adjusted for baseline macular thickness (MT). All analyses initially considered any RD/breaks as the event, followed by rhegmatogenous RD/breaks.

**Results:**

The mean (SD) baseline age was 54.8 (8.2) years. Sixty-four participants developed RD/breaks (of any subcategory), with a mean (SD) of 7.0 (3.3) years between baseline and disease onset. A more negative baseline FRO was independently associated with an increased risk of any RD/breaks (adjusted hazard ratio [HR] = 0.66, 95% confidence interval [CI] = 0.50–0.87, *P* = 0.003) and rhegmatogenous RD/breaks (HR = 0.61, 95% CI = 0.45–0.82, *P* = 0.001). Similar independent associations were evident in the subgroup analysis that additionally adjusted for MT.

**Conclusions:**

A more negative baseline FRO is associated with a higher risk of developing RD/breaks, even among individuals with similar baseline SER and other risk factors. This demonstrates a potential benefit of shifting towards an anatomic definition of myopia.

That high myopia is a significant risk factor for rhegmatogenous retinal detachment (RD) is supported by a strong evidence base. At the population level, conventional descriptors of myopia—namely spherical equivalent refraction (SER) and axial length (AL)—have been shown to exhibit strong associations with the risk of RD.^1^^–^^7^

Both SER and AL are, however, ultimately one-dimensional and on-axis (point to point along the visual axis) summaries of ocular dimensions, limiting their ability to capture the anatomic peculiarities across an individual's posterior eye. This may partly explain why, at the individual level, the risks of myopic complications, including RD, can still vary significantly even when myopia severity is similar.^6^^–^^9^ One example of the limitation of SER as an on-axis descriptor of ocular dimensions is that, while the posterior vertex of the globe becomes more curved (less oblate or more prolate) with decreasing SER, substantial variations in posterior eye shape can still be observed among eyes with similar SER.^10^ We previously showed that some of these variations, uncaptured by SER, could be captured by variations in certain fundus features.^10^

To address this limitation, we have recently proposed a metric to characterize ametropia at a more personalized, “anatomic” level (Fig. 1): fundus refraction offset (FRO).^11^ For an eye with a given SER, a more negative FRO suggests a more myopic-looking fundus than typical for that SER, which is analogous to how a more positive retinal age gap suggests an “older-looking” fundus than one's chronological age.^12^ We previously demonstrated that a more negative FRO was associated with lower macular thickness (MT) and reduced choroidal vascularity index, even among individuals with similar SER or AL, as well as age, sex, and ethnicity. In this longitudinal analysis of participants in the UK Biobank cohort, we aimed to investigate whether baseline FRO provided any additional value in stratifying the risk of future RD/breaks, beyond what existing risk factors, particularly baseline SER, could provide. We hypothesized a negative association between baseline FRO and future disease risk, even after adjusting for baseline SER and other relevant covariates.

**Figure 1.**
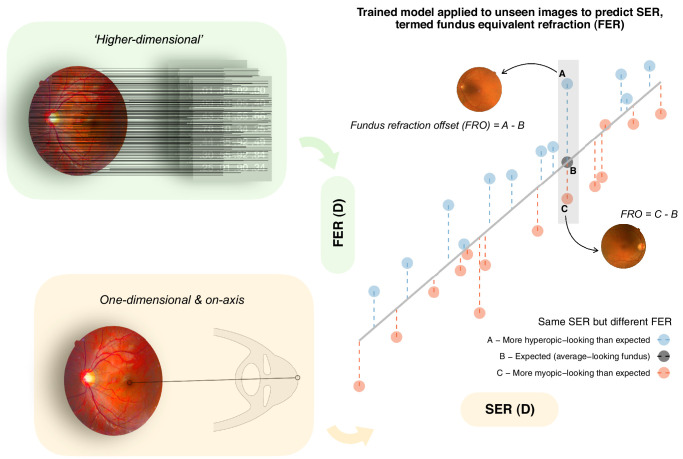
Overview of fundus refraction offset (FRO). A deep learning model was previously trained on fundus images from phakic eyes without any posterior eye conditions to predict spherical equivalent refraction (SER), with the expectation that the trained model—having achieved an optimal bias-variance trade-off (good generalizability to unseen images)—would have learned to capture the non-pathological variations in fundus appearance across a broad spectrum of SER.^11^ When the trained model is applied to unseen images, the fundus-predicted SER is termed fundus equivalent refraction (FER) to distinguish it from SER in the strictly one-dimensional and on-axis sense. As FER is derived directly from pixel-level information across an individual's fundus, it is thought to contain “higher-dimensional” information about the eye, capturing more subtle interindividual differences in posterior segment anatomy not otherwise fully captured by SER, as demonstrated in our recent work.^11^ To derive FRO, the deviation of each observation from the line of best fit (i.e. residuals from linear regression) is computed, as visually represented by the *vertical dashed lines* in the scatterplot. This is illustrated using three examples with the same SER but different FER: the eye that lies on the line (datapoint B) shows zero deviation from the norm; the eye that shows positive deviation (datapoint A) has a relatively hyperopic fundus appearance; and the eye that shows negative deviation (datapoint C) has a relatively myopic fundus appearance.

## Methods

### Description of the UK Biobank Cohort

The UK Biobank is a large-scale prospective cohort comprising approximately half a million adults aged 40 to 69 years, recruited from 3 constituent countries of the United Kingdom: England, Scotland, and Wales.^13^ Extensive assessments were carried out at baseline between 2006 and 2010, including self-administered questionnaires on sociodemographic information and health/medical history, face-to-face interview with a study nurse, and physical examination. The physical examination included anthropometric measurements using the Tanita BC418MA Body Composition Analyzer (for weight) and the Seca 202 stadiometer (for height).

Ophthalmic assessments with retinal imaging were subsequently introduced between 2009 and 2010.^14^ During this period, a total of 67,321 participants attending the baseline visit additionally underwent a 45-degree macula-centered fundus photography (Topcon 3D OCT-1000 Mark II), non-cycloplegic autorefraction (Tomey RC-5000), and distance visual acuity (VA) testing (Precision Vision digital LogMAR chart). As the UK Biobank has Research Tissue Bank approval from the Northwest Multi-Center Research Ethics Committee (06/MRE08/65), separate ethical clearance was not required for this study.

### Longitudinal Health-Related Data Sources

Participants were invited to follow-up visits every few years to repeat the baseline assessments, including the self-administered questionnaires. Additionally, a wide range of health outcomes were captured longitudinally and regularly through linkages to routinely available national health-related data sources. These included national death registries, with data available from 2006 to 2022 for all 3 UK constituent countries, as well as primary care records (1938–2016 for England, 1939–2017 for Scotland, and 1948–2017 for Wales) and hospital admission/operation records (1981–2022 for Scotland, 1991–2022 for Wales, and 1997–2022 for England) from the National Health Service—the UK's comprehensive and universal healthcare system free at the point of use.

Using these linked real-world data sources, along with participants’ self-reported health/medical history at each assessment visit, a wide range of ocular and systemic conditions were mapped to three-digit International Classification of Diseases (ICD-10) codes.^15^ Data fields recording the date of first occurrence of each condition were then generated (hereafter referred to as the “first occurrence” data). In this study, we analyzed the data collected during the baseline assessment visit as well as the longitudinal first occurrence data.

### Eligibility Criteria

The eligibility criteria were similar to those applied in our recent work.^11^ Specifically, included eyes must meet the following criteria at baseline: fundus photographs of adequate image quality, as determined by a validated deep learning model^16^ (excluded if classified as “reject” quality; examples are shown in Supplementary Fig. S1), presence of refractive error data, no prior history of refractive/cataract surgery, good VA (≤ 0.00 LogMAR), and no prior history of posterior eye conditions. Posterior eye conditions included chorioretinal disorders (such as RD/breaks and pathologic myopia), scleral or globe disorders, glaucoma, and non-glaucomatous optic neuropathy. Readers are referred to our previous publication for additional details, including a comprehensive description of these conditions.^11^

### Independent Variable, Event of Interest, and Right Censoring

The independent variable, FRO, was derived from individual-level errors in SER predicted from fundus images using deep learning (termed fundus equivalent refraction [FER]), which is detailed in Figure 1 and in our previous work.^11^ The event of interest, RD/breaks, was defined at the individual level (ICD-10 linkages were specific to participants rather than eyes) using the first occurrence data.^15^ Participants with a recorded entry of ICD-10 code “H33,” which encompassed a broad category of RD/breaks, were identified, along with the date of the first occurrence.

The various subcategories of RD/breaks classified by ICD-10 were also ascertained by accessing the individual records from which the first occurrence data were sourced, for each participant with a recorded entry of H33. These subcategories included rhegmatogenous RD (H33.0), retinoschisis and retinal cysts (H33.1), serous retinal detachment (H33.2), retinal breaks without detachment (H33.3), traction detachment of the retina (H33.4), and other retinal detachments (H33.5).

In the survival analyses described below, all subcategories of RD/breaks were initially considered collectively as the event of interest. After this, only rhegmatogenous RD (H33.0) and retinal breaks (H33.3) were considered, as these subcategories have a more direct pathophysiological link to myopia. The right-censoring date was May 31, 2022, or earlier if a participant had died or was lost to follow-up (left the United Kingdom or withdrew from follow-up).

### Covariates

Covariates including SER, age, sex, ethnicity, Townsend deprivation index, cataract surgery during follow-up, history of ocular trauma/injury, diabetes, hypertension, and body mass index (BMI) were selected a priori. These covariates were specific to the baseline visit—except for “cataract surgery during follow-up,” which was a Boolean variable indicating whether a participant had undergone cataract surgery (or some form of lens extraction) during follow-up prior to RD/breaks onset or the right-censoring date (whichever came earlier). This information was sourced from hospital records by identifying relevant procedural codes classified by the Office for Population Census and Survival Classification of Surgical Operations Version 4, namely C71 (extracapsular extraction of lens), C72 (intracapsular extraction of lens), and C74 (other extraction of lens).

Ethnicity was analyzed as a binary variable, categorizing participants as either White or non-White, given the predominance of White ethnicity in the UK Biobank. History of ocular trauma/injury was inferred from participants’ response to the question, “What was your age when injury or trauma resulting in loss of vision was first diagnosed,” analyzed as a Boolean variable (positive history if there was a response). Diabetes (ICD-10: E10 to E14) and systemic hypertension (ICD-10: I10) were also analyzed as Boolean variables, indicating if a participant had a recorded entry on or before the baseline visit based on the first occurrence data.^15^ FRO, SER, age, Townsend deprivation index (where more positive values corresponded to greater material deprivation),^17^ and BMI (calculated as weight [kg] divided by the square of height [m]) were treated as continuous variables unless stated otherwise.

### Overall and Subgroup Survival Analyses

Kaplan-Meier survival curves were first generated to visualize the unadjusted survival probability, that is, the probability of remaining RD/breaks-free, over time for participants with different baseline categories of FRO: > +1.00 diopter (D), +0.50 to +1.00 D, 0 to +0.49 D, −0.50 to −0.01 D, −1.00 to −0.49 D, and < −1.00 D. Cox regression was then used to assess the univariable (unadjusted) associations of baseline FRO (as a continuous variable) and several covariates with survival time. The independent (adjusted) association between FRO and survival time was then tested by including FRO, SER, age, sex, and other covariates that showed evidence of a univariable association with survival time in a single multivariable Cox regression model. As a subgroup analysis, we also examined whether the association between FRO and RD/breaks risk was independent of baseline MT across the whole Early Treatment of Diabetic Retinopathy Study region—because retinal thinning may have relevance to the propensity of RD/breaks in myopia—in a subset of participants with optical coherence tomography (OCT) scans that passed quality control (as previously detailed in Ref. 11).

Where both eyes of a participant were eligible, their SER, FER, and MT were averaged across both eyes in all analyses described above, as the event of interest was defined at the individual level and there were very strong inter-eye linear correlations for these variables (Supplementary Figs. S2–S4). Multicollinearity for each variable in multivariable Cox regression was assessed using the variance inflation factor (VIF), which suggested little evidence of multicollinearity (VIF ≤ 1.15 for all variables in all models).^18^

### Sensitivity Analyses

Previous studies have demonstrated the ability of deep learning to predict age from fundus photographs. To rule out the possibility that FRO might have somehow relied on complex (nonlinear) information pertinent to age,^12^^,^^19^ the multivariable Cox regression mentioned above was repeated twice—first, after including a quadratic age term, *Age + Age^2^* (sensitivity analysis 1a), and then after including both quadratic and cubic terms, *Age + Age^2^ + Age^3^* (sensitivity analysis 1b). To rule out the influence of refractive ametropia, multivariable Cox regression was also repeated after excluding eyes with absolute cylindrical power of 2D or higher (sensitivity analysis 2). In another sensitivity analysis, we excluded cases sourced exclusively from self-reported information (sensitivity analysis 3).

Missing data were handled using complete case analysis in all instances. All statistical tests were 2-tailed, with *P* < 0.05 considered evidence of a statistical association. R version 4.2.2 (R Core Team 2022, r-project.org) was used to perform all analyses, and the source code is openly available at github.com/fyii200/FundusRefractionOffset.

## Results

### Participant Characteristics

Of the 67,321 participants attending the baseline visit between 2009 and 2010, 31,068 participants without any prior history of RD/breaks met the eligibility criteria. However, a random subset of these participants (70%) had previously been used to train the deep learning model for predicting FER (from which FRO was derived). Therefore, the remaining 9320 participants, which were never used for model training, were included in the overall survival analysis. Of these, 7127 participants had OCT scans that passed quality control and were included in the subgroup survival analysis.

Table 1 summarizes the baseline characteristics of these participants. Overall, the mean baseline age, SER and FRO were 54.8 years, −0.22 D and 0.00 D, respectively, with 91.6% being White and 54.1% female participant. “Indian/Pakistani” (*n* = 176), “Caribbean” (*n* = 130), “Other ethnic group” (*n* = 123), and “African” (*n* = 115) were the largest non-White ethnicities in the dataset. A total of 512 participants underwent cataract surgery during follow-up. Only data for Townsend deprivation index and BMI were missing (in 18 and 49 participants, respectively).

**Table 1.** Baseline Characteristics of Participants Included in the Overall Analysis

	Overall	No RD	RD Onset
Characteristics^*^	(*n* = 9320)	(*n* = 9256)	(*n* = 64)
Age, y	54.8 (8.2)	54.8 (8.2)	56.5 (6.9)
Female, *n* (%)	5046 (54.1)	5021 (54.2)	25 (39.1)
White, *n* (%)	8539 (91.6)	8478 (91.6)	61 (95.3)
SER, D	−0.22 (2.20)	−0.22 (2.20)	−1.19 (2.59)
FRO, D	0.00 (0.69)	0.00 (0.69)	−0.25 (1.03)
Townsend	−1.13 (2.92)	−1.13 (2.92)	−0.84 (3.18)
Cataract surgery^†^	512 (5.5)	501 (5.4)	11 (17.2)
Time from baseline to cataract surgery, y	7.7 (4.3)	7.7 (4.3)	7.0 (2.9)
Diabetes, *n* (%)	355 (3.8)	351 (3.8)	4 (6.3)
Hypertension, *n* (%)	2085 (22.4)	2069 (22.4)	16 (25.0)
BMI, kg/m^2^	27.1 (4.6)	27.1 (4.6)	27.9 (4.9)
Self-reported history of ocular trauma/injury, *n* (%)	44.0 (0.5)	44 (0.5)	0 (0.0)

BMI, body mass index; FRO, fundus refraction offset; RD, retinal detachment or breaks; SER, spherical equivalent refraction.

* Mean (SD) reported for continuous variables.

† During follow-up prior to RD/breaks onset or right-censoring date.

### Number and Overall Incidence of RD/Break Cases

The mean (SD) follow-up duration was 11.9 (1.3) years, with 414 and 22 participants having a right-censoring date before May 31, 2022, due to death and loss to follow-up, respectively. During this period, 64 participants (0.69%) developed RD/breaks (any subcategory of ICD-10 H33), with a mean (SD) of 7.0 (3.3) years between baseline and disease onset. All 64 cases were sourced from hospital records, except for 2, where 1 case was sourced from primary care records, and another was based on self-reported health/medical history. Information on the various subcategories of RD/breaks was available for the 62 cases sourced from hospital records. Among these, 35 were rhegmatogenous RD and 11 were retinal breaks (Supplementary Fig. S5 provides further details). Compared with participants who did not develop RD/breaks, those who developed RD/breaks (any subcategory) had more myopic baseline SER (−1.19 D versus −0.22 D) and FRO (−0.25 D versus 0.00 D), on average (see Table 1). However, none of the 44 participants with a self-reported history of ocular trauma/injury prior to the baseline visit developed RD/breaks, so the association of ocular trauma/injury with RD/breaks could not be tested in the analyses below.

### Baseline FRO Negatively and Independently Associated With Disease Risk

**Figure 2.**
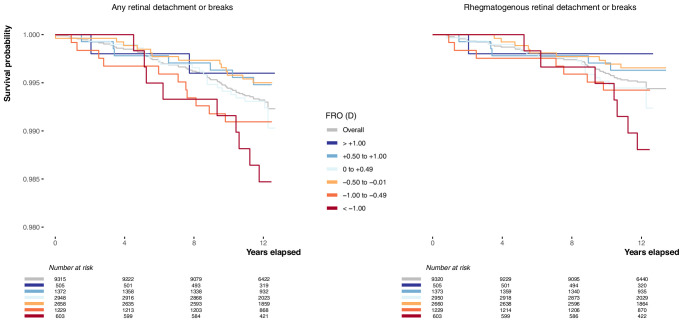
Kaplan-Meier survival curves showing the unadjusted survival probability, that is, the probability of remaining event-free over time, in participants with different baseline categories of FRO. Event is defined as any subcategory of retinal detachment or retinal breaks on the left, whereas on the right only rhegmatogenous retinal detachment or retinal breaks. “Number at risk” indicates the number of participants that remain event-free or have not been right-censored at each time point.

Figure 2 shows the Kaplan-Meier survival curves for different baseline categories of FRO. Overall (unstratified by baseline FRO), approximately 0.7% (1 – survival probability) of participants developed any subcategory of RD/breaks and 0.5% developed rhegmatogenous RD/breaks after 11.5 years. However, among participants with the most myopic baseline FRO, the proportions were 1.5% for any RD/breaks and 1.2% for rhegmatogenous RD/breaks after 11.5 years—representing 1.7-fold and 2.4-fold increases in event rate, respectively, relative to the unstratified figures.

Using univariable Cox regression models, we found that a more negative baseline SER, a more negative baseline FRO, male sex, and cataract surgery during follow-up were associated with an increased risk (hazards) of RD/breaks (Table 2). Importantly, the association between baseline FRO and RD/breaks risk remained significant even after adjusting for baseline SER, age, sex, and cataract surgery during follow-up using multivariable Cox regression.

**Table 2.** Univariable (Unadjusted) and Multivariable (Adjusted) Associations of FRO and Other Covariates With RD/Breaks Using Cox Regression in the Overall Analysis of 9320 Participants

	**Any RD/Breaks**				**Rhegmatogenous RD/Breaks**			
Variable^*^	Unadjusted HR (95% CI)	*P* Value	Adjusted HR (95% CI)	*P* Value	Unadjusted HR (95% CI)	*P* Value	Adjusted HR (95% CI)	*P* Value
SER	0.85	**<** **0** **.001**	0.84	**<** **0** **.001**	0.81	**<** **0** **.001**	0.80	**<** **0** **.001**
	(0.78–0.93)		(0.77–0.92)		(0.74–0.90)		(0.72–0.89)	
FRO	0.65	**0** **.002**	0.66	**0** **.003**	0.61	**0** **.001**	0.61	**0** **.001**
	(0.50–0.86)		(0.50–0.87]		(0.45–0.81)		(0.45–0.82)	
Age	1.03	0.07	1.03	0.12	1.02	0.38	1.02	0.41
	(1.00–1.06)		(0.99–1.06)		(0.98–1.05)		(0.98–1.06)	
Male	1.86	**0** **.02**	1.84	**0** **.02**	2.47	**0** **.004**	2.47	**0** **.004**
	(1.13–3.08)		(1.11–3.05)		(1.33–4.57)		(1.33–4.58)	
Cataract surgery^†^	3.56	**<** **0** **.001**	2.99	**0** **.002**	3.06	**0** **.006**	2.74	**0** **.02**
	(1.86–6.81)		(1.50–5.97)		(1.37–6.85)		(1.16–6.43)	
White	1.87	0.29	/	/	2.02	0.33	/	/
	(0.59–5.95)				(0.49–8.32)			
Townsend (18 missing)	1.03	0.41	/	/	1.04	0.37	/	/
	(0.95–1.12)				(0.95–1.15)			
Diabetes	1.75	0.28	/	/	1.82	0.32	/	/
	(0.64–4.81)				(0.57–5.87)			
Hypertension	1.17	0.58	/	/	0.74	0.44	/	/
	(0.67–2.07)				(0.35–1.59)			
BMI (49 missing)	1.04	0.15	/	/	1.02	0.54	/	/
	(0.99–1.09)				(0.96–1.08)			

HR, hazard ratio.

The left column treats all subcategories of RD/breaks as the event of interest, whereas the right column focuses solely on rhegmatogenous RD/breaks as the event of interest. Statistically significant *p*-values are shown in bold.

* All variables are specific to the baseline visit except cataract surgery.

† During follow-up prior to RD/breaks onset or right-censoring date.

Specifically, an adjusted hazard ratio (HR) of 0.66 in the multivariable model that considered any subcategory of RD/breaks as the event of interest indicated that, among eyes with similar baseline SER and other covariates, the risk of RD/breaks was 1.52-fold (inverse of 0.66) and 2.27-fold (inverse of 0.66^2^) higher for every −1.00 D and −2.00 D difference in baseline FRO, respectively (compared with 1.25-fold and 1.56-fold higher risk for equivalent differences in baseline SER). Baseline FRO decreased the Akaike information criterion (AIC; a lower value indicated a more parsimonious, i.e. better, model)^20^ by 5.6 and increased the concordance index (C-index; a value closer to one indicated superior discriminative ability)^21^ from 0.675 to 0.690.

This independent effect of baseline FRO appeared somewhat stronger (lower HR) when the event was defined as rhegmatogenous RD/breaks, with an adjusted HR of 0.61. Baseline FRO decreased the AIC by 6.7 and increased the C-index from 0.701 to 0.717.

### Effect of Baseline FRO on Disease Risk Similarly Independent of Baseline MT

Table 3 shows the multivariable Cox regression results for the subgroup of participants with good-quality OCT data. As before, a more negative baseline FRO was associated with a higher RD/breaks risk—even after adjusting for baseline MT, baseline SER, and other covariates—with a stronger association noted when considering only rhegmatogenous RD/breaks as the event of interest (HR = 0.55) compared with any RD/breaks (HR = 0.68).

**Table 3.** Multivariable (Adjusted) Associations of FRO and Other Covariates—Including Baseline Macular Thickness—With RD/Breaks Using Cox Regression in the Subgroup Analysis of 7127 Participants

	Any RD/Breaks		Rhegmatogenous RD/Breaks	
Variable^*^	Adjusted HR (95% CI)	*P* Value	Adjusted HR (95% CI)	*P* Value
SER	0.85 (0.75–0.95)	**0** **.007**	0.79 (0.69–0.90)	**<** **0** **.001**
FRO	0.68 (0.46–1.00)	**0** **.049**	0.55 (0.36–0.82)	**0** **.004**
Age	1.02 (0.98–1.06)	0.27	1.01 (0.96–1.05)	0.78
Male	1.33 (0.73–2.42)	0.35	1.56 (0.76–3.20)	0.22
Cataract surgery^†^	2.22 (0.84–5.88)	0.11	2.08 (0.60–7.22)	0.25
Macular thickness	1.01 (0.98–1.03)	0.61	1.01 (0.98–1.03)	0.66

The left column treats all subcategories of RD/breaks as the event of interest, whereas the right column focuses solely on rhegmatogenous RD/breaks as the event of interest. Statistically significant *p*-values are shown in bold.

* All variables are specific to the baseline visit except cataract surgery.

† During follow-up prior to RD/breaks onset or right-censoring date.

### Sensitivity Analyses

Table 4 presents the adjusted HR for baseline FRO across the sensitivity analyses described above. The inclusion of nonlinear age terms—quadratic and/or cubic (sensitivity analyses 1a and 1b)—did not affect the independent association between baseline FRO and the risk of RD/breaks. Likewise, evidence of a significant association remained after excluding eyes with high cylindrical power (sensitivity analysis 2) and after removing one self-reported case (sensitivity analysis 3).

**Table 4.** Multivariable Association of Baseline FRO With any RD/Breaks and Rhegmatogenous RD/Breaks in the Following Sensitivity Analyses: 1a (Including a Quadratic Age Term), 1b (Including Quadratic and Cubic Age Terms), 2 (Excluding 305 Eyes With Absolute Cylindrical Power of 2D or Higher), and 3 (Excluding One Self-Reported Case)

	Any RD/Breaks		Rhegmatogenous RD/Breaks	
Sensitivity Analysis	Adjusted HR (95% CI)	*P* Value	Adjusted HR (95% CI)	*P* Value
1a	0.66 (0.50–0.86)	**0** **.002**	0.61 (0.46–0.82)	**0** **.001**
1b	0.61 (0.46–0.82]	**0** **.001**	0.61 (0.46–0.82)	**0** **.001**
2	0.64 (0.49–0.85)	**0** **.002**	0.59 (0.43–0.79)	**<** **0** **.001**
3	0.67 (0.51–0.89)	**0** **.005**	0.61 (0.45–0.82)	**0** **.001**

Statistically significant *p*-values are shown in bold.

## Discussion

In this 12-year prospective study of mid-life adults with no prior history of posterior eye conditions, a more negative FRO at baseline—indicating a relatively more myopic-looking fundus—was independently associated with a higher risk of future RD/breaks. Specifically, the adjusted HR from the multivariable Cox regression in Table 2 suggests that the risk is 1.52-fold (any RD/breaks) and 1.64-fold (rhegmatogenous RD/breaks) higher for every −1 D difference in baseline FRO, even among individuals with similar baseline SER and other relevant covariates. Likewise, in the subgroup analysis of participants with good-quality OCT data, a similar independent association was observed while additionally adjusting for baseline MT. Although the independent association of FRO was evident across all analyses—whether the event was defined as any RD/breaks or only rhegmatogenous RD/breaks—the association appeared stronger for rhegmatogenous RD/breaks.

The pathogenesis of RD is well documented.^22^^–^^24^ One key precursor to the onset of both rhegmatogenous and tractional RD is the tractional forces acting on the neurosensory retina. In rhegmatogenous RD, retinal breaks produced and maintained by vitreoretinal tractional forces allow fluid from liquefied vitreous to enter the subretinal space, precipitating detachment of the neurosensory retina from the underlying retinal pigment epithelium. Myopia is thought to increase the risk of rhegmatogenous RD through its association with vitreous liquefaction.^24^ In tractional RD, on the other hand, the tractional forces arise from contractile fibrous or fibrovascular tissue, such as that typically seen in neovascular conditions like proliferative diabetic retinopathy.

It is possible that these tractional forces, even in the early stages, lead to subtle morphologic and geometric changes in fundus imaging features—such as vessel tortuosity and concavity of vascular arcades (the extent to which the superior and inferior vessels converge towards the macula). In an at-risk eye, these changes may, in turn, give rise to a fundus appearance that deviates significantly from that typical of a relatively healthy eye with the same SER, which is what FRO is designed to capture. Such a biomechanical (traction-induced) view of fundus changes would be consistent with myopic changes in fundus features—including vessel tortuosity, concavity of vascular arcades, and neuroretinal rim pallor—which can be attributed to biomechanical (ocular stretching) mechanisms,^25^^–^^28^ although changes in certain dimensional fundus features may additionally have an optical origin.^29^ In serous RD, however, such fundus changes may not manifest as early due to the absence of a tractional component.^22^ This may explain why baseline FRO appeared more strongly associated with rhegmatogenous RD/breaks than when considering all subcategories of RD/breaks collectively as the event of interest (serous RD accounted for 18% of all cases, as shown in Supplementary Fig. S5).

In addition to increased vitreous liquefaction leading to larger tractional forces acting on the neurosensory retina, myopia may also increase RD risk through its direct structural impact on the retina^22^^,^^30^: in everyday clinical language, the retina is not uncommonly described as “more stretched” in myopia.^31^ Specifically, during myopic expansion of the globe, the retina is subjected to stress imposed by the changing shape of the much stiffer outer layers of the eye,^30^ particularly the sclera.^32^ Because the expansion of the globe is fundamentally a three-dimensional process, the accompanying impact on the retina or fundus cannot be fully captured by SER or AL alone, given their one-dimensional and on-axis nature (see Fig. 1).^10^ For example, two different eyes with similar SER or AL can still differ in their posterior vertex curvature, and thus have different fundus appearance—a difference that FRO likely better captures owing to its “higher-dimensional” nature, as it is directly derived from pixel-level information across the fundus (see Fig. 1).

Among the various covariates examined in this study, a more negative baseline SER, male sex, and cataract surgery during follow-up were all independently associated with an increased risk of RD/breaks—whereas other baseline variables, such as age, BMI, and hypertension, were not associated with risk. Our findings for SER and cataract surgery are consistent with well-known epidemiological associations reported in the literature.^1^^,^^9^^,^^33^^–^^35^ The positive association between male sex and risk is commonly reported in studies analyzing samples that underwent cataract surgery.^35^^,^^36^ In the general population, male subjects also generally appear to be at higher risk, although whether this is attributable to an inherent biological difference or sex-related risk of ocular trauma/injury remains unclear.^37^ Although the incidence of rhegmatogenous RD generally increases with age,^37^ due to age-related vitreous liquefaction,^38^ the lack of such an association in the present study may be due to the relatively narrow age range (40–69 years old) with no younger or very old adults serving as comparators. Finally, whether systemic factors are associated with RD risk remains unclear. For instance, in contrast to the positive association between BMI and rhegmatogenous RD risk found in a case-control study,^39^ we found insufficient longitudinal evidence of such an association—a null finding similarly reported by a retrospective analysis of longitudinal data from a large cohort of male Swedish conscripts.^40^ The authors of that study, however, found an increased risk of rhegmatogenous RD among hypertensive individuals.^40^

### Limitations

Despite its strength as a large-scale, population-based prospective cohort study with health outcome data captured in real time (or closer to real time than if health outcomes were only captured at regular follow-up visits) through data linkages to the UK's universal healthcare system, the present study has several limitations. First, the lack of AL information precluded further investigation into the AL-adjusted association of baseline FRO with the risk of RD/breaks. Although baseline FRO is still likely to have an independent association with risk because it does not merely reflect a mismatch between SER and AL (FRO was previously found to be associated with OCT-derived parameters even after AL adjustment),^11^ this association may be somewhat weaker after accounting for AL. Second, the generalizability of our findings to non-White populations and to real-world clinical settings—where fundus photographs are not necessarily macula-centered with a 45-degree field of view—remains to be validated. Likewise, the model's generalizability to different imaging systems (non-Topcon fundus cameras) and its robustness to noise arising from media opacities require further evaluation. Third, the lack of laterality information meant that the data could only be analyzed at the individual level, potentially resulting in some loss of statistical power. However, this is very unlikely to have biased the results, given the strong inter-eye correlation observed for the eye-specific variables analyzed. Finally, we were unable to investigate the effect of ocular trauma/injury due to the absence of RD/break cases with a positive history. We suspect that some participants either provided unreliable responses or had relatively inconsequential ocular trauma/injury, as despite reporting a positive history resulting in “loss of vision,” all of them had good baseline VA (0.00 LogMAR or better). In summary, external validation utilizing a diverse range of datasets sourced from other populations and settings, along with the inclusion of AL measurements, would strengthen the conclusions of this study.

## Conclusions

A more negative baseline FRO—indicating a more myopic fundus than typical for a given SER—is associated with a higher risk of developing RD/breaks over 12 years, even among individuals with similar baseline SER and other relevant risk factors in our sample of UK Biobank participants. This demonstrates the potential of FRO as a personalized risk biomarker for RD/breaks, which may be valuable in facilitating a more targeted approach to patient management or education (e.g. on the importance of seeking immediate medical attention if relevant symptoms occur)^41^ in primary care. Likewise, FRO could enhance the personalization of risk-benefit assessment for elective refractive surgeries. For example, between two equally myopic patients, corneal refractive surgery might be a safer option than refractive lens exchange (higher RD risk)^42^ for the individual with a more markedly negative FRO.

A broader and more important implication of FRO is the paradigm shift toward an anatomic “view” or definition of myopia (or ametropia generally),^10^^,^^11^^,^^25^^,^^43^ consistent with the repositioning of myopia as a disease with clinical phenotypic (anatomic) alterations.^44^^,^^45^ Because the ultimate goal of myopia control is to minimize the long-term risks of pathological sequalae, a point worth pondering^43^^,^^46^ is whether the anatomic severity of myopia, rather than SER or AL alone, should ultimately be what we aim to control.

## Supplementary Material

Supplement 1
