# Supplementary material for: Fundus Refraction Offset as a Personalized Biomarker for 12-Year Risk of Retinal Detachment
Source: Invest Ophthalmol Vis Sci. 2025 Jul 1;66(9):1. doi: 10.1167/iovs.66.9.1 (PMC12227021; doi:10.1167/iovs.66.9.1)

Supplementary Figure S1: Examples of fundus photographs automatically rejected due to poor image quality.

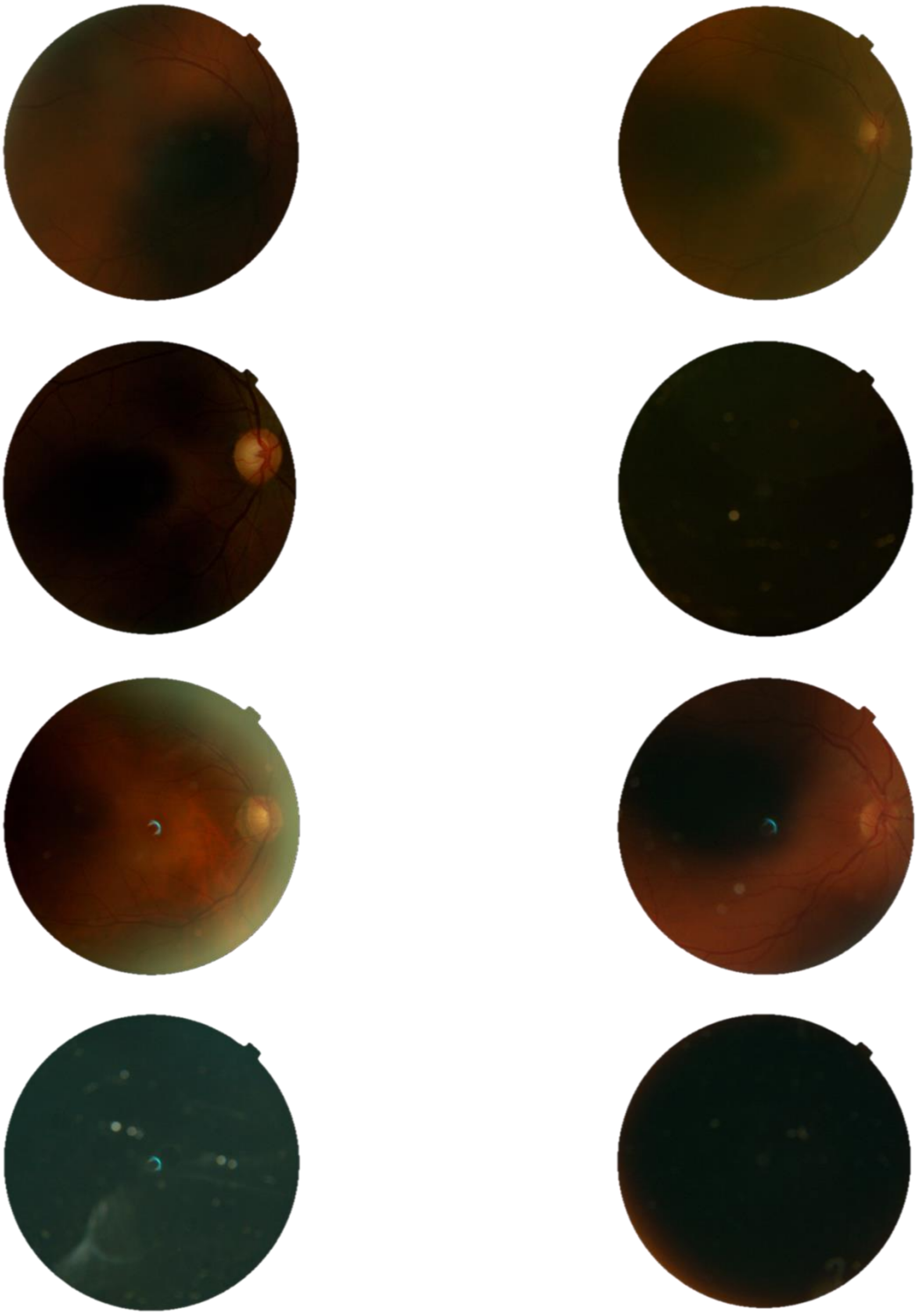

Supplementary Figure S2: Strong linear correlation of spherical equivalent refraction (SER) between eyes. Data from both eyes of 4,190 participants in the overall analysis were averaged.

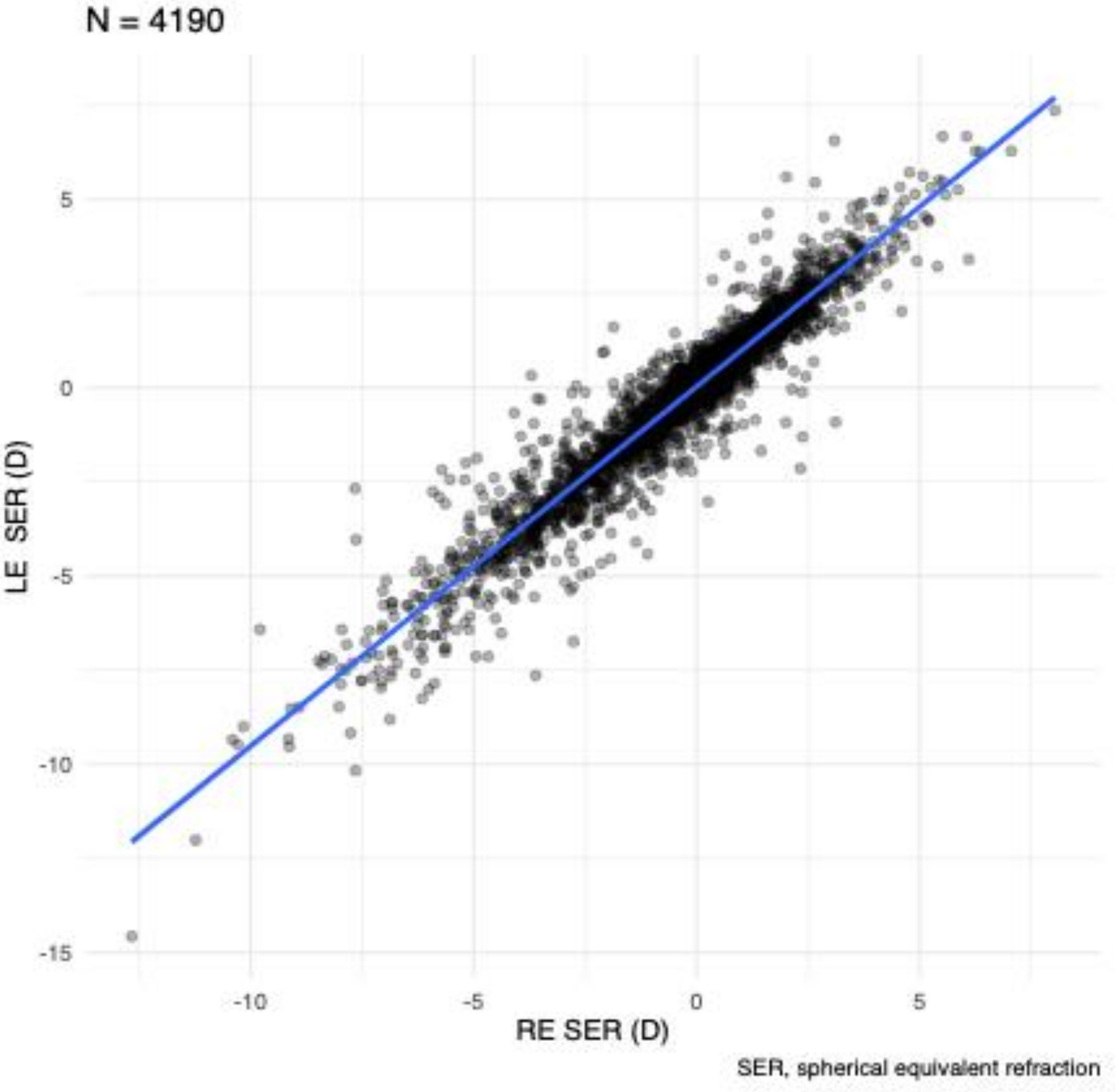

Supplementary Figure S3: Strong linear correlation of fundus equivalent refraction (FER) between eyes. Data from both eyes of 4,190 participants in the overall analysis were averaged.

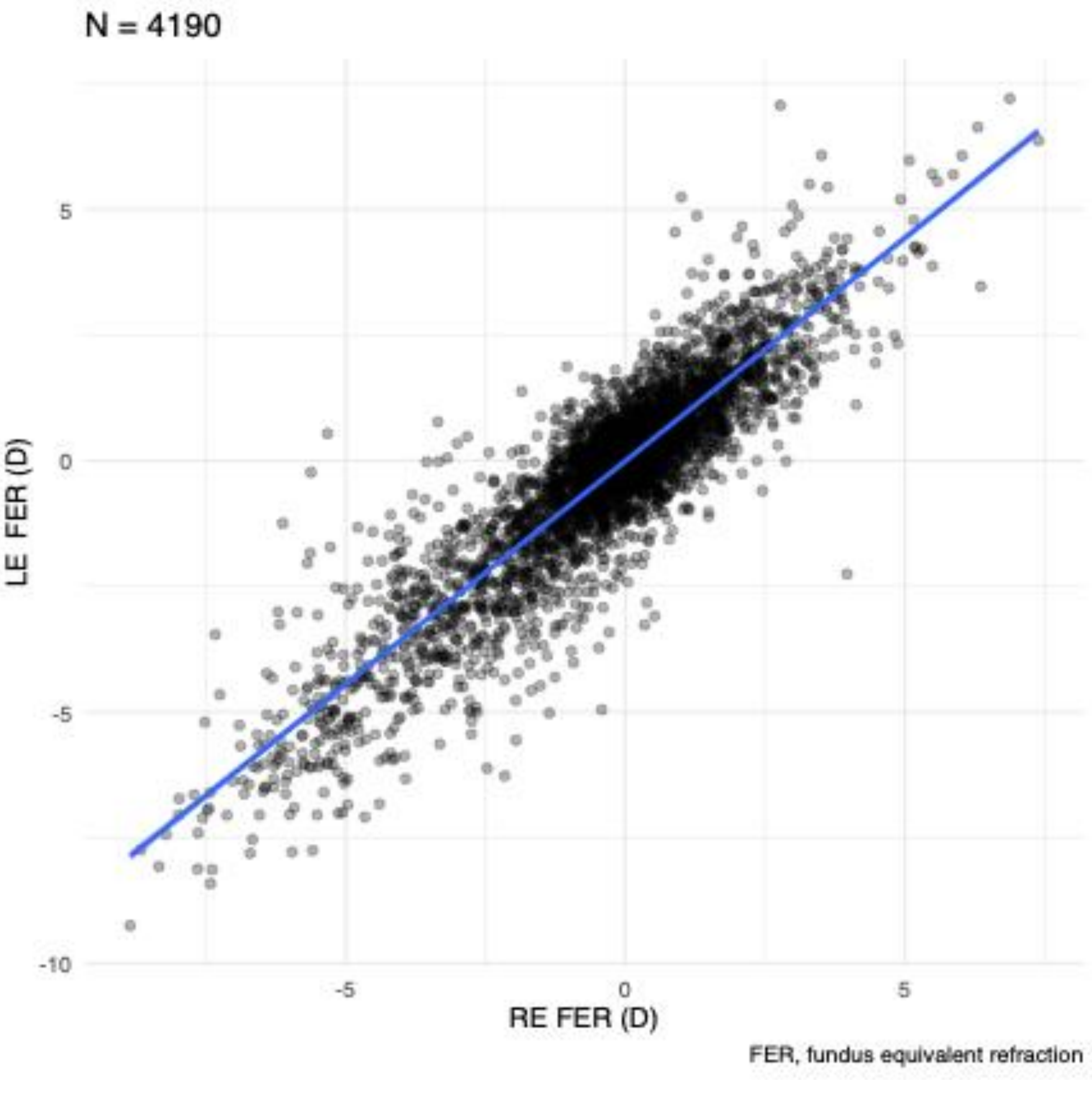

Supplementary Figure S4: Strong linear correlation of baseline macular thickness (MT) between eyes. Data from both eyes of 2,397 participants were averaged in the subgroup analysis.

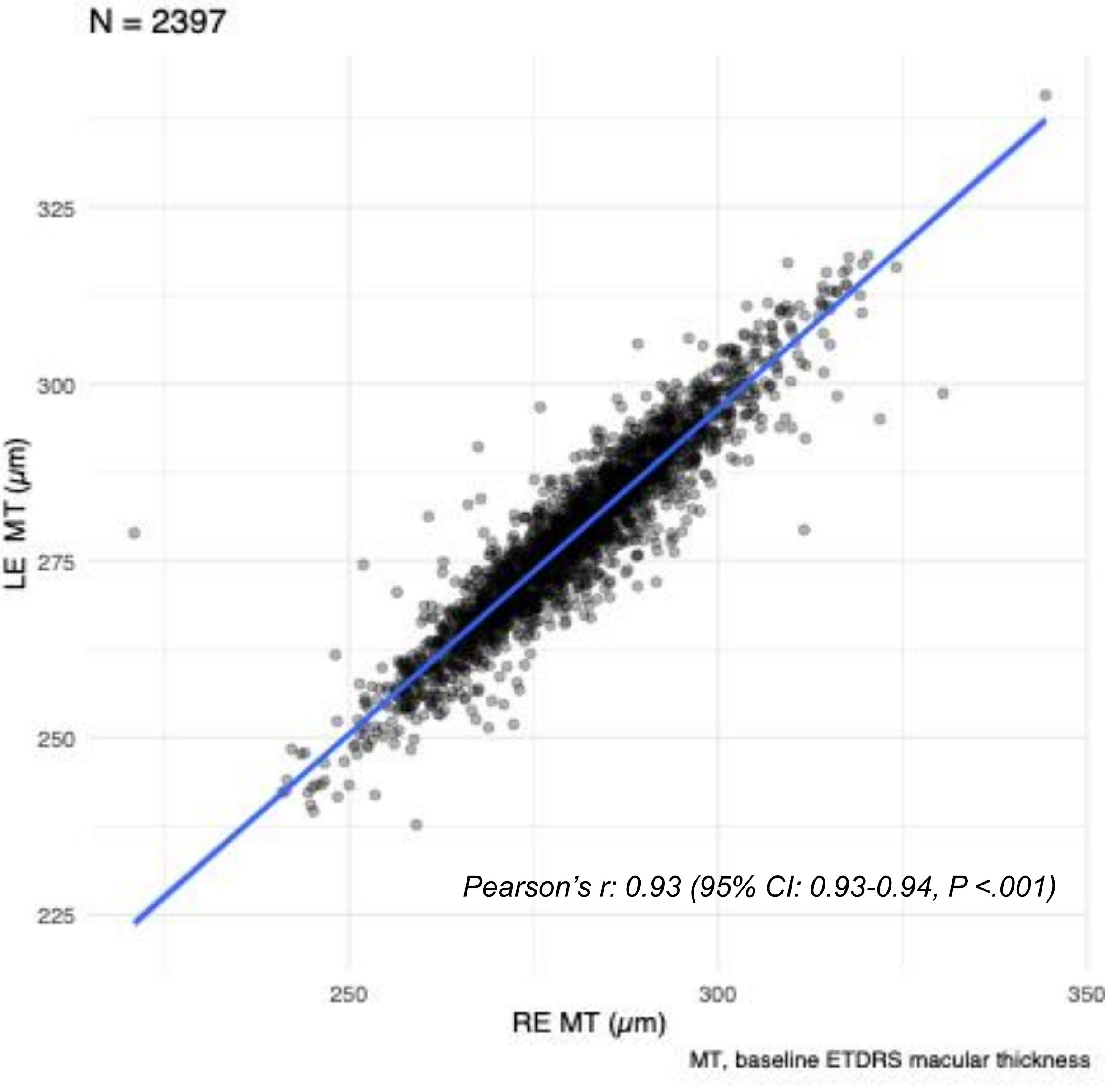

Supplementary Figure S5: Number and proportion of newly onset 'retinal detachments (RD) and breaks', as classified by the 10th Revision of the International Classification of Diseases (ICD-10) and sourced from hospital records.

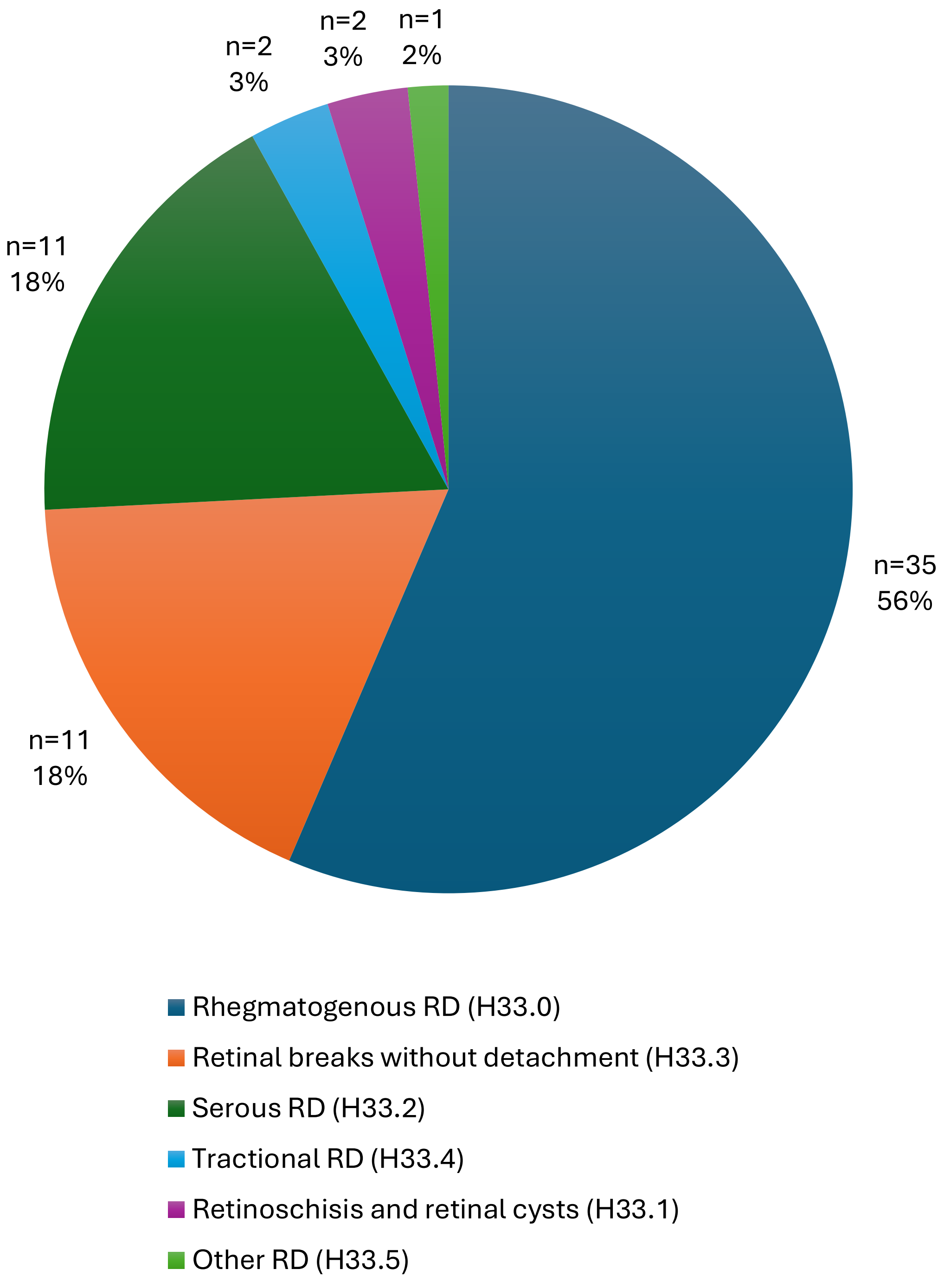

Supplement: Supplement 1 [file iovs-66-9-1_s001.pdf]
